# A Rice Immunophilin Homolog, OsFKBP12, Is a Negative Regulator of Both Biotic and Abiotic Stress Responses

**DOI:** 10.3390/ijms21228791

**Published:** 2020-11-20

**Authors:** Ming-Yan Cheung, Wan-Kin Auyeung, Kwan-Pok Li, Hon-Ming Lam

**Affiliations:** 1 School of Life Sciences, The Chinese University of Hong Kong, Shatin, N.T., Hong Kong SAR; cheungmy@cuhk.edu.hk (M.-Y.C.); hknavyfield@yahoo.com.hk (W.-K.A.); likwanpok108080@hotmail.com (K.-P.L.); 2 Center for Soybean Research of the State Key Laboratory of Agrobiotechnology, The Chinese University of Hong Kong, Shatin, N.T., Hong Kong SAR

**Keywords:** biotic stress, abiotic stress, immunophilin, FKBP, salt tolerance, OsYchF1, rice, suppression subtractive hybridization

## Abstract

A class of proteins that were discovered to bind the immunosuppressant drug FK506, called FK506-binding proteins (FKBPs), are members of a sub-family of immunophilins. Although they were first identified in human, FKBPs exist in all three domains of life. In this report, a rice *FKBP12* homolog was first identified as a biotic stress-related gene through suppression subtractive hybridization screening. By ectopically expressing *OsFKBP12* in the heterologous model plant system, *Arabidopsis thaliana*, for functional characterization, OsFKBP12 was found to increase susceptibility of the plant to the pathogen, *Pseudomonas syringae* pv. *tomato* DC3000 (*Pst* DC3000). This negative regulatory role of FKBP12 in biotic stress responses was also demonstrated in the *AtFKBP12-*knockout mutant, which exhibited higher resistance towards *Pst* DC3000. Furthermore, this higher-plant FKBP12 homolog was also shown to be a negative regulator of salt tolerance. Using yeast two-hybrid tests, an ancient unconventional G-protein, OsYchF1, was identified as an interacting partner of OsFKBP12. OsYchF1 was previously reported as a negative regulator of both biotic and abiotic stresses. Therefore, OsFKBP12 probably also plays negative regulatory roles at the convergence of biotic and abiotic stress response pathways in higher plants.

## 1. Introduction

FK506-binding proteins (FKBPs) belong to a sub-family of immunophilins. Immunophilins (including FKBPs and cyclophilins) were first described as the receptors of immunosuppressant drugs used in clinical settings to prevent graft rejection in transplantation [1]. For instance, the first discovered FKBP in human, hFKBP12, was shown to be the receptor for rapamycin and the synthetic immunosuppressant drug, FK506 [2]. FKBPs have been identified in all three domains of life and contain a conserved functional domain annotated with peptidyl-prolyl cis-trans isomerase (PPIase) activities (EC 5.2.1.8). This domain was thought to catalyze the cis/trans isomerization of proline residues in a polypeptide. Such activities were largely demonstrated in vitro and were thought to be associated with protein folding and maturation [3,4].

FKBPs are involved in many signal transduction pathways and cellular regulatory systems [3,5]. In animals, 7 and 18 FKBPs were discovered in *Drosophila melanogaster* and *Homo sapiens* respectively [6]. Members of the FKBP sub-family vary in molecular sizes, numbers of the conserved FKBP domain, presence/absence of other functional domains/motifs and presence/absence of targeting signals. Human FKBP12 was shown to be a cell cycle regulator through its interactions with different proteins, including calcium-binding proteins, transforming growth factor and FKBP12-rapamycin binding proteins [7]. However, similar studies in plants were limited. The genome annotations of *Arabidopsis thaliana*, *Oryza sativa* and *Zea mays* reveal a rich collection of FKBPs (23 in Arabidopsis, 29 in rice and 30 in maize) in different cellular compartments, with a primary focus on plastid-localized FKBPs [5,6,8]. While some studies of plant FKBPs focused on reproducing their molecular functions already discovered in mammalian systems [9,10], other studies have shown that FKBPs participated in novel functions in plants. FKBP members of various sizes were reported in *A. thaliana* to be involved in cell differentiation, heat stress, redox signaling, auxin transport and photosystem assembly [11,12,13,14,15,16].

Many plant FKBPs were thought to play roles as foldases, by maintaining proper protein folding during stress responses [17]. However, increasing evidence has led to a suggestion by a recent review that PPIase-like proteins might actually modify proteins involved in pathogenesis [18]. For instance, the Arabidopsis cyclophilin, ROC1, could recognize type III effectors of the pathogen *Pseudomonas syringae* [19,20], and activate effector-triggered immunity, illustrating the potential role of immunophilins as molecular switches in defense signaling. One of the Arabidopsis *FKBP* members, *AtFKBP65* (At5g48570) was reported to be induced upon pathogen (*Pseudomonas syringae* and *Xanthomonas campestris*) inoculation [21]. However, transgenic Arabidopsis over-expressing the cDNA of this gene showed no difference in defense response toward *Xanthomonas campestris* invasion [21]. *PaFKBP12* from *Polytrichastrum alpinum* (alpine haircap moss) collected in the Antarctic was implicated in development and stress responses. Its expression could be induced by heat and abscisic acid. Transgenic Arabidopsis ectopically expressing *PaFKBP12* is larger in size, a result of increased rates of cell cycle. In addition, *PaFKBP12* ectopic expressors exhibited enhanced tolerance towards salt and drought stresses [22].

On the other hand, AtFKBP12 was shown to interact with an ATPase, AtFIP37, in vitro, which was proposed to participate in RNA splicing [11,14]. While mutation studies suggested AtFIP37 to be related to trichrome development, the role of AtFKBP12 was not addressed. However, this clearly suggested that FKBPs could interact with nucleotide-binding proteins.

In this study, we conducted rapid functional tests of a rice immunophilin homolog (*OsFKBP12*) in transgenic *A. thaliana* to show that OsFKBP12 was involved in both biotic and abiotic stress responses. Protein-protein interaction studies identified the unconventional GTPase, OsYchF1, also involved in both biotic and abiotic stress responses, as one of the interacting partners of OsFKBP12.

## 2. Results

### 2.1. Identification of OsFKBP12, a Gene Encoding an FK506-Binding Protein (FKBP) in Oryza sativa

**Figure 1:**
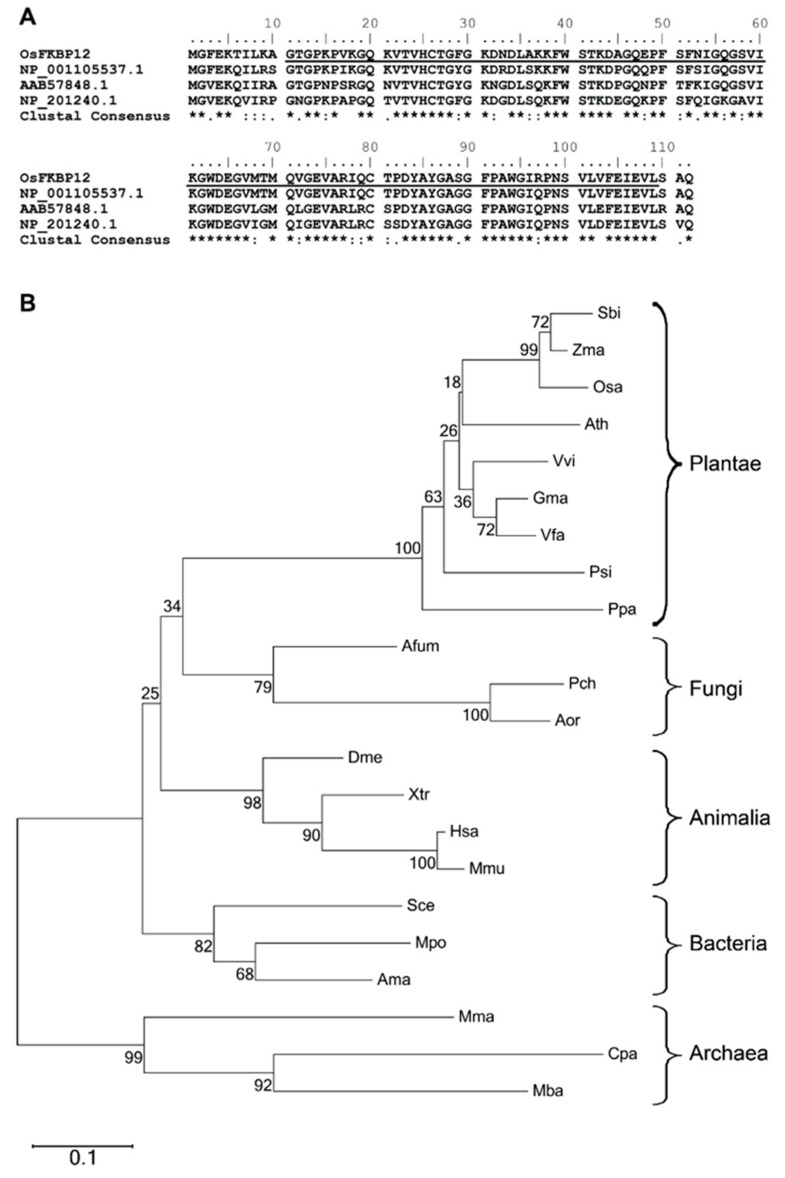
(**A**) Alignment of the OsFKBP12 protein sequence with homologs in *Z. mays* (NP_001105537), *V. faba* (AAB57848) and *A. thaliana* (NP_201240) illustrating the presence of the conserved FK506-binding protein (FKBP)-specific domain. Amino acid residue alignment was performed by ClustalW [24]. “*”: conserved residues; “:”: conserved substitutions; and “.”: semi-conserved substitutions. The putative position of the FKBP domain identified in the Conserved Domain Database (CDD) is underlined. (**B**) Phylogenetic analysis of FKBP12-like proteins from representative genomes from different kingdoms. The tree was constructed using the neighbor-joining method. Bootstrap values are indicated. Sbi, *Sorghum bicolor* (XP_002454586); Zma, *Z. mays* (NP_001105537); Osa, *O. sativa* (NP_001048188); Ath, *A. thaliana* (NP_201240); Vvi, *Vitis vinifera* (XP_002263647); Gma, *Glycine max* (ACU15318); Vfa, *V. faba* (AAB57848); Psi, *Picea sitchensis* (ABK22086); Ppa, *Physcomitrella patens* subsp. patens (XP_001756105); Afum, *Aspergillus fumigatus* Af293 (XP_751096); Pch, *Penicillium chrysogenum* Wisconsin 54-1255 (XP_002559257); Aor, *Aspergillus oryzae* RIB40 (BAE64049); Dme, *Drosophila melanogaster* (NP_523792); Xtr, *Xenopus tropicalis* (XP_002944067); Hsa, *Homo sapiens* (NP_000792); Mmu, *Mus musculus* (NP_032045); Sce, *Sorangium cellulosum* ‘So ce 56′ (CAN93956); Mpo, *Methylobacterium populi* BJ001 (ACB83281); Ama, *Acaryochloris marina* MBIC11017 (ABW26812); Mma, *Methanoculleus marisnigri* JR1 (ABN57243); Cpa, *Candidatus Parvarchaeum acidiphilum* ARMAN-4 (EEZ92550); Mba, *Methanosarcina barkeri* str. Fusaro (YP_305460).

We initially obtained the partial cDNA clone of *OsFKBP12* as part of a previous study [23], using the suppression subtractive hybridization technique with RNA samples from a pair of near-isogenic rice lines (NILs), either the NIL CBB14 which contains the *R* gene *Xa14* or its susceptible recurrent parent (SN1033), at 4 days post-inoculation (dpi) with the pathogen *Xanthomonas oryza* pv. *oryza* (*Xoo*). Here we focused on characterizing the functions of this gene as it relates to defense response and stress tolerance. The nucleotide sequence of the full-length cDNA clone is 100% identical to an annotated rice (*Oryza sativa*) cDNA sequence directly deposited in the public database (GenBank accession: NM_001054723), and encodes a protein with 112 amino acid residues (Figure 1A). The corresponding gene in the rice genome is designated as a single-copy gene located on chromosome 2 (Gene: Os02g0760300). BlastP analysis revealed that the translated peptide from our clone is identical to a putative immunophilin in the rice genome (GenBank accession: NP_001048188), with 89%, 76% and 74% amino acid identity to clones in *Zea mays* (NP_001105537), *Vicia faba* (AAB57848) and *A. thaliana* (NP_201240) respectively (Figure 1A). All of them are annotated as FKBP (FK506-binding protein)-type peptidyl-prolyl cis-trans isomerases (PPIases). Hence, we named our clone *OsFKBP12* to reflect its homology to other FKBPs according to its putative FKBP-specific domain and its size, following the conventional naming system of the FKBP sub-family. Figure 1A shows the alignment of *OsFKBP12* with its homologs from *Z. mays*, *V. faba* and *A. thaliana*. Of particular note is that the amino acid sequences of these FKBPs were almost entirely composed of the underlined FKBP domain.

BlastP search revealed that FKBP12-like proteins existed in all kingdoms of life. After sequence alignment, a phylogenetic tree was built via neighbor-joining method with 1000 bootstrap replicates, using sequences from representative members of each kingdom with available genomic data in GenBank (Figure 1B). Bacterial and archaeal FKBP12-like proteins are variable in sequence lengths, from 109 up to 170 amino acid residues. On the other hand, within the respective kingdoms themselves, plant and animal FKBP12 homologs were highly similar in sequence lengths (comprising 112 and 109 amino acid residues for plant and animal FKBP12s, respectively), with high bootstrap values. Within the kingdom Plantae, FKBPs could be further grouped into monocots (e.g., *Sorghum bicolor*, *Zea mays* and *Oryza sativa*) and dicots (e.g., *Arabidopsis thaliana*, *Vitis vinifera*, *Glycine max* and *Vicia faba*).

### 2.2. OsFKBP12 Expression was induced by the Pathogen Xanthomonas oryza pv. oryza (Xoo) in the Susceptible Recurrent Parents of Resistant NILs

**Figure 2:**
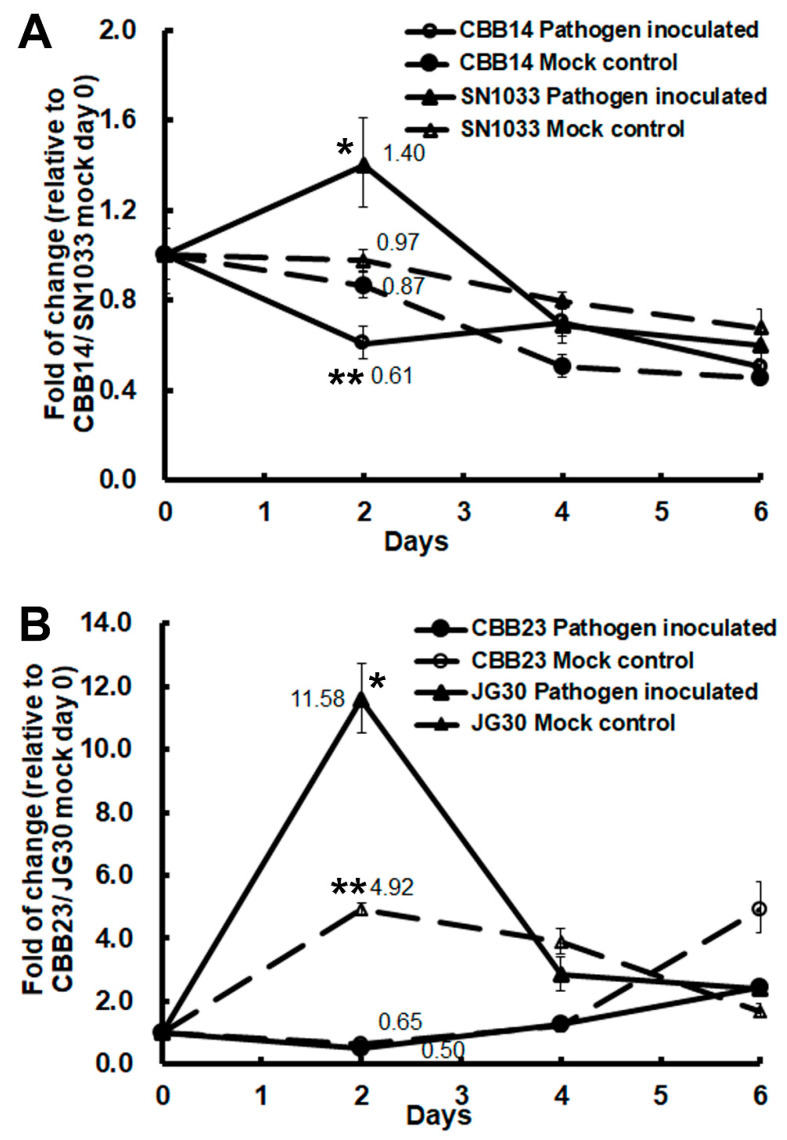
Expression studies of *OsFKBP12* in bacterial blight-resistant near-isogenic lines (NILs) and their respective susceptible recurrent parents. (**A**) Expressions of *OsFKBP12* in the resistant NIL (CBB14, carrying the *Xa14 R* locus) and its susceptible recurrent parent (SN1033) of the Japonica variety, upon the challenge with *Xanthomonas oryza* pv. *oryza* (*Xoo*) race LN44. (**B**) Expressions of *OsFKBP12* in the resistant NIL (CBB23, carrying the *Xa23 R* locus) and its susceptible recurrent parent (JG30), which is an Indica variety, upon the challenge with *Xoo* race P6. The 8-week-old plants were inoculated with *Xoo* (pathogen inoculated) or water (mock control) by a clipping method. Day-0 leaf samples were collected before inoculation. Leaf tissues about 6–8 mm away from the inoculation sites were collected 2 and 4 days after inoculation. Total RNA samples were prepared from the leaf tissues collected, reverse-transcribed, and subjected to real-time PCR. Relative gene expression was calculated by the 2^−∆∆CT^ method [25] and normalized against the expression of the rice actin gene, *OsAc1D* [26]. Fold-of-change in *OsFKBP12* expression in each treatment was compared to the expression on mock control day 0. Folds of change in gene expressions on day 2 are indicated with numerical data. Solid diamond: CBB14/CBB23-pathogen inoculated; open diamond: CBB14/CBB23-mock control; solid triangle: SN1033/JG30-pathogen inoculated; open triangle: SN1033/JG30-mock control. The statistical significance of the differences between day 2 and day 0 for each treatment of each line were analyzed by one-way ANOVA analysis, followed by LSD as post-hoc test. “*” indicates *p* < 0.05; “**” indicates *p* < 0.005. The error bars indicate standard deviation (*n* = 3).

We wanted to see if the expression of *OsFKBP12* is affected by encounters with bacterial blight, so we inoculated two resistant-NIL-and-susceptible-recurrent-parent pairs: CBB14 (carrying the *Xa14 R* locus) and its susceptible recurrent parent (SN1033 of the Japonica variety), and CBB23 (carrying the *Xa23 R* locus) and its susceptible recurrent parent (JG30, Indica variety), with *Xoo* race LN44 and *Xoo* race P6, respectively. In both cases, *OsFKBP12* was significantly induced in the susceptible recurrent parents (SN1033 and JG30) upon virulent *Xoo* inoculation but not in their corresponding resistant offspring lines (Figure 2). On the contrary, a significant repression of *OsFKBP12* was observed in the *Xoo*-resistant line CBB14. These results implicate the possibility of OsFKBP12 playing a negative role in pathogenesis.

### 2.3. Ectopic Expression of OsFKBP12 Repressed Salicylic Acid (SA)-Related Defense Marker Genes and Enhanced the Susceptibility to Pseudomonas syringae pv. Tomato DC3000 (Pst DC3000) in Transgenic A. thaliana

Based on the presence of the conserved FKBP domain and its identification from a defense-related subtractive cDNA library, we hypothesized that the OsFKBP12 protein is a component of a common plant defense signaling pathway against pathogens. A rapid functional test was performed in the heterologous model plant system, *A. thaliana*. *OsFKBP12* was constitutively expressed in *A. thaliana* ecotype Col-0 driven by the Cauliflower Mosaic Virus (CMV) 35S promoter via *Agrobacterium*-mediated transformation. Three independent homozygous lines with a single insertion locus were traced (lines F, G and J). Two Arabidopsis *AtFKBP12*-knockout mutant lines (CS849010 and SALK_064494) and the ecotype Col-2 (wild-type background of the *Atfkbp12* mutant, CS849010) were also included in the functional study. The expression of the *OsFKBP12* transgene in the transgenic lines and *AtFKBP12* in the two mutant lines were verified by reverse transcription followed by real-time PCR. The expression level of *OsFKBP12* in line J was the lowest and was set to 1 for comparison of the transgene expression levels (Figure S1). No signal for *OsFKBP12* was obtained for Col-0 after prolonged PCR amplification, and no signal for *AtFKBP12* was detected in the two Arabidopsis mutant lines, either.

**Figure 3:**
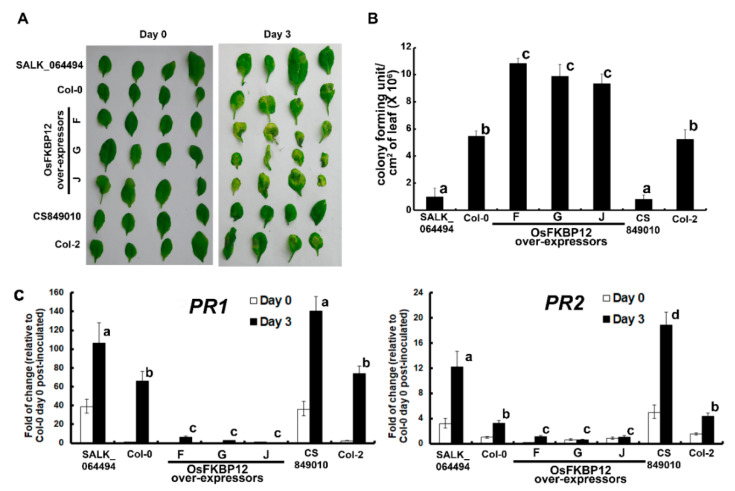
Pathogen inoculation of *OsFKBP12*-transgenic *A. thaliana*. Three independent transgenic Arabidopsis lines (F, G and J) ectopically expressing *OsFKBP12*, two *AtFKBP12-* knockout mutant lines of Arabidopsis (CS849010 and SALK_064494) and two wild-types (Col-0 and Col-2) were inoculated with the pathogen, *Pseudomonas syringae* pv. *tomato* (*Pst* DC3000), at six weeks old by syringe infiltration at a concentration of 10^8^ colony forming units/mL in 10 mM MgSO_4_ supplemented with 0.02% (*v*/*v*) Silwet L-77 [27]. Leaves were harvested three days post-inoculation for (**A**) phenotypic analyses, (**B**) pathogen titer determination and (**C**) relative gene expression analyses of two defense marker genes, *PR1* and *PR2*. The pathogen titers (colony-forming units per unit area) at the inoculation zones (harvested as leaf discs) were estimated by plate count method [27]. Relative gene expressions were calculated using the 2^−∆∆CT^ method, with *UBQ10* as the normalizing gene [28,29]. For fold-of-change calculations, the expression levels of *PR1* and *PR2* in Col-0 on day 0 were set to 1 as reference. Statistical significance was analyzed using one-way analysis of variance combined with the Games–Howell post hoc test. Error bars represent standard errors. Different lowercase letters above the bars represent statistically distinct groups at *p* < 0.05. Two biological repeats with similar results were obtained.

To clearly demonstrate the relationship of over-expressing *OsFKBP12* and disease susceptibility, a pathogen inoculation test using the biotrophic bacterium, *Pseudomonas syringae* pv. *tomato* DC3000 (*Pst* DC3000), was carried out on transgenic *A. thaliana* overexpressing *OsFKBP12* and the two *Atfkbp12* mutant lines. The development of disease symptoms on inoculated leaves, the pathogen titers at the inoculation zones and the expression patterns of defense marker genes (*PR1* and *PR2*) were assessed (Figure 3). Compared to Col-0, more severe yellowing and necrosis could be observed on inoculated leaves of the *OsFKBP12*-transgenic lines, while the two *Atfkbp12* mutant lines had alleviated symptoms (Figure 3A). In addition, significantly higher titers of the pathogen than wild-type controls were recorded at the inoculation zones of *OsFKBP12-*transgenic lines and significantly lower pathogen titers than controls were found in the two *Atfkbp12* mutant lines (Figure 3B). Despite some induction of *PR1* and *PR2* expressions resulting from pathogen inoculation in the *OsFKBP12*-transgenic lines, their expression levels were substantially lower than those in the wild-types Col-0 and Col-2, and the two *Atfkbp12* mutant lines had significantly higher expressions of these two genes than their corresponding wild-type controls (Figure 3C).

### 2.4. Ectopic Expression of OsFKBP12 in Arabidopsis Increased Salt Sensitivity and Reduced Expressions of Genes Related to Salt Tolerance

**Figure 4:**
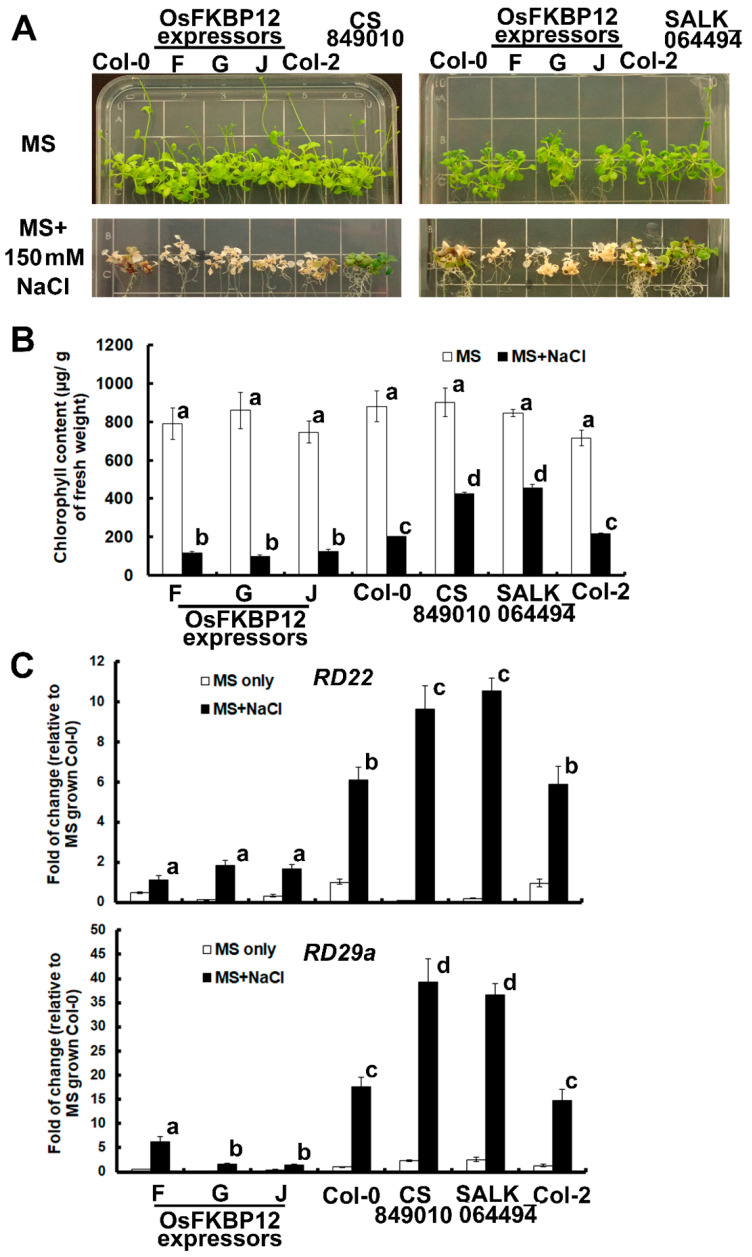
Salt treatment of *OsFKBP12-*transgenic *A. thaliana*. Ten-day-old seedlings grown on MS agar plates were transferred onto MS plates supplemented with 150 mM NaCl (salt treatment) or MS-only plates (mock treatment). Phenotypes of salt stress were recorded after 10 days of treatment. (**A**) Leaf chlorosis was severe in the three OsFKBP12-expressors, and *AtFKBP12-*knockout mutant lines (CS849010 and SALK_064494) exhibited very minor leaf chlorosis, while wild-type lines (Col-0 and Col-2) were somewhere in between in terms of stress phenotype. (**B**) Chlorophyll contents were determined to quantify the degree of leaf chlorosis. (**C**) The expressions of salt-inducible genes (*RD22* and *RD29a*) were obtained from samples harvested after 1 day of salt treatment. Relative gene expressions were calculated using the 2^−∆∆CT^ method, with *UBQ10* as the normalizing gene [28,29]. The expression levels of *RD22* and *RD29a* in Col-0 under mock treatment were set to 1 for reference. Statistical significance was analyzed using one-way analysis of variance combined with the Games–Howell post hoc test. Error bars represent standard errors with at least three biological replicates. Different lowercase letters above the bars represent statistically distinct groups at *p* < 0.05. Two biological repeat experiments were conducted with similar results.

As FKBP12 was reported to confer tolerance for abiotic stresses including salt and drought in alpine haircap moss [22], we next examined the effect of overexpressing *OsFKBP12* in transgenic Arabidopsis under salt treatment, and compared that to the two Arabidopsis knockout mutant lines. For this purpose, 10-day-old seedlings grown on MS agar plates were transferred to MS agar supplemented with 150 mM NaCl or MS agar only (mock treatment). Phenotypes were observed after 10 days of treatment, and chlorophyll contents were measured to quantify the extent of chlorosis. Meanwhile the expressions of salt-inducible genes (*RD22* and *RD29a*) were determined in leaf samples harvested 1 d after treatment. Figure 4A shows the phenotypes of salt- and mock-treated *OsFKBP12*-transgenic Arabidopsis and the two *Atfkbp12* mutant lines. It was apparent that the three transgenic lines with OsFKBP12 ectopic expression (F, G and J) exhibited severe salt damage with comparatively more extensive leaf chlorosis. On the other hand, the two *Atfkbp12* mutants showed the least amount of leaf chlorosis, while wild-type lines (Col-0 and Col-2) displayed intermediate levels of salt damage phenotype. Chlorophyll content was measured to show the effects of salt treatment quantitatively (Figure 4B). Two well-known salt-inducible genes, *RD22* and *RD29a*, were used as indicators to demonstrate the molecular effects of ectopically expressing *OsFKBP12* when the transgenic plants were under salt treatment. Basal expressions of the two salt-inducible genes in all three transgenic lines were lower than the expression in the wild-types. Upon salt treatment, significantly lower induction was found in all three *OsFKBP12*-transgenic lines compared to the two wild-types, which in turn had significantly lower induction than the *Atfkbp12* mutants (Figure 4C).

### 2.5. OsFKBP12 Protein Interacted with an Ancient Unconventional G-Protein, OsYchF1

To identify the interacting partners of OsFKBP12 hypothesized in a potential general defense signaling cascade, two rounds of yeast two-hybrid screening were performed. The *OsFKBP12* clone was subcloned into the yeast expression vector pGBKT7 to be fused to the DNA-binding domain of GAL4 as bait and transformed into the yeast strain Y187. Successful production of the fusion protein from the yeast cells was confirmed by western blot using anti-cMyc antibodies. The prey library in yeast was constructed previously using the yeast expression vector pGADT7-Rec and transformed into the yeast strain AH109. This library was used in previous studies to identify interacting protein partners [23,30,31].

The library screening was done by mating Y187, containing pGBKT7-*OsFKBP12*, with AH109, containing the cDNA library. By selecting for the three reporter genes and viable growth on selective media, and blue color development in the colony-lift assay, one cDNA clone representing a partial fragment of a putative protein was identified (Tables S2 and S3). The plasmids containing the partial cDNA clone were extracted and purified. Then these plasmids were co-transformed into AH109 together with pGBKT7-*OsFKBP12*. The results from yeast two-hybrid screening on selective media and colony-lift color assay were consistent with each other (Figure S2).

To identify the interacting protein partner, the amino acid sequences of this unknown protein were translated by the ExPASy Translate tool. BlastP searches in public genome databases were performed and the sequences were matched to an annotated protein accession BAD03576 deposited in GenBank. The partial fragment that matched BAD03576 represents 41 amino acid residues of that accession, with an overall sequence coverage of 10.3%.

BAD03576 in fact represents OsYchF1, a putative G-protein containing a YchF domain that was shown to be a negative regulator of rice defense responses [32]. Its coding sequence is located on chromosome 8 in the rice genome. BAD03576 has a homolog in Arabidopsis called AtYchF1 (GenBank accession: NP_174346), which shares 85% amino acid sequence identity with the rice version.

To verify the results from the yeast two-hybrid experiments, in-vitro pull-down assays were performed. Full-length cDNAs of *OsFKBP12*, *OsYchF1* and *AtYchF1* were sub-cloned into the *E. coli* expression vector pGEX-4T-1, which adds an in-frame *N*-terminal glutathione-S-tranferase (GST) tag to the proteins expressed [23,32]. Using the *E. coli* expression vector pMAL-c2, OsFKBP12 fusion protein containing an in-frame *N*-terminal Maltose Binding Protein (MBP) tag was also constructed. The fusion proteins were expressed in *E. coli* strain DE3/BL21. Successful expression events were confirmed by resolving the protein bands on SDS-PAGE after a purification step using the appropriate protein purification kits and detected by Coomassie Brilliant Blue staining.

**Figure 5:**
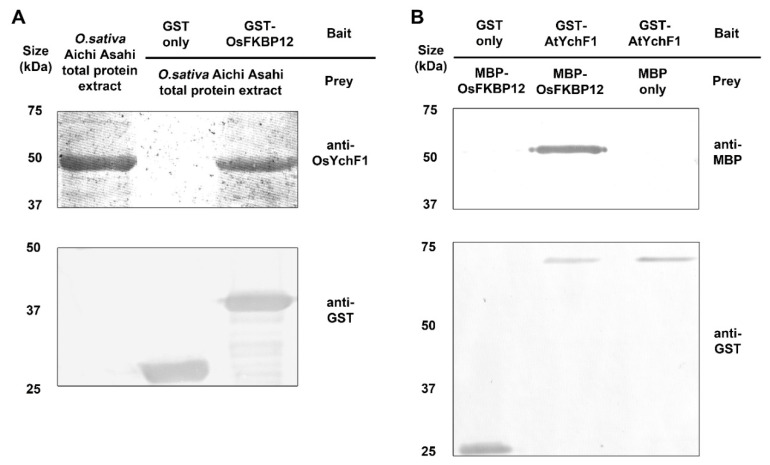
In-vitro pull-down experiments of OsFKBP12 with YchF1 homologs from rice and Arabidopsis. (**A**) In-vitro pull-down of OsFKBP12 with the native unconventional G-protein OsYchF1. Around 500 μg total protein extracted from Aichi Asahi rice strain was mixed with 100 μg GST-tagged proteins or GST tag only. The MagneGST^TM^ Glutathione Particles were used for pulling down the protein complex, followed by western blot detection using anti-OsYchF1 antibodies and anti-GST antibodies. (**B**) In-vitro pull-down of OsFKBP12 with the Arabidopsis homolog of OsYchF1, AtYchF1. Around 100 μg cell lysate containing GST-tagged proteins were mixed with 100 μg cell lysate containing MBP-tagged proteins. The MagneGST^TM^ Glutathione Particles were used for pulling down the protein complex, followed by western blot detection using anti-MBP and anti-GST antibodies. MBP tag alone and GST tag alone were used as negative controls to confirm the specificity of the interactions. The experiment was performed twice with similar results.

To confirm the interaction between OsFKBP12 and OsYchF1, GST-OsFKBP12 was incubated with crude rice protein extract from the Japonica cultivar Aichi Asahi, and the MagneGST^TM^ Glutathione Particles were used for pulling down the protein complex containing GST-OsFKBP12 and its interacting proteins. Western blot analysis of the resulting protein complex using antibodies against OsYchF1 confirmed the interaction between OsFKBP12 and OsYchF1 (Figure 5A). A negative control using the GST tag protein alone did not pull down OsYchF1. The interaction between OsFKBP12 and OsYchF1 supported the result of yeast two-hybrid screening.

To see if OsFKBP12 can also interact with AtYchF1 as additional evidence to support the results from the functional studies using the heterologous transgenic Arabidopsis system, an in-vitro pull-down experiment was performed to detect the interaction between OsFKBP12 and AtYchF1, using purified MBP-OsFKBP12 and purified GST-AtYchF1, and MagneGST^TM^ Glutathione Particles were used for pulling down the protein complex. Western blot analysis using anti-GST antibodies and anti-MBP antibodies to detect GST-AtYchF1 and MBP-OsFKBP12 respectively in the protein complex confirmed the interaction between OsFKBP12 and AtYchF1 (Figure 5B). A negative control using the MBP tag protein alone did not pull down GST-AtYchF1. In addition, bimolecular fluorescence complementation (BiFC) experiment was conducted to evaluate in vivo interaction between OsFKBP12 and OsYchF1. Fusion constructs containing *nYFP-OsFKBP12* and *cCFP-OsYchF1* were expressed in protoplasts prepared from tobacco BY-2 suspension cells via PEG-mediated transformation. Negative controls with protoplasts expressing *nYFP-OsFKBP12* + *cCFP* and *cCFP-OsYchF1* + *nYFP* construct pairs were also examined to demonstrate the specific interaction between OsFKBP12 and OsYchF1 (Figure S3). Fluorescent signals could only be observed in protoplasts co-expressing *nYFP-OsFKBP12* and *cCFP-OsYchF1*.

## 3. Discussion

### 3.1. OsFKBP12 Encodes an FKBP (FK506-Binding Protein) with a Conserved FKBP-Specific Domain in Oryza sativa

The *OsFKBP12* clone was discovered by differential expression in the defense-related subtractive cDNA library. Similar to its homologs in *Z. mays*, *V. faba* and *A. thaliana*, the conserved FKBP domain encompassed nearly the entire protein sequence (Figure 1A). The FKBP domain was characterized as being able to catalyze the slow cis/trans isomerization of proline residues within a polypeptide [4]. Compared to the extensive studies on mammalian FKBPs due to the interests in drug targets and functions, plant FKBPs, on the other hand, were rarely examined. FKBP domain-containing proteins of various sizes were reported in *A. thailiana* to be involved in cell differentiation, heat stress, redox signaling, auxin transport and photosystem assembly [11,12,13,14,15,33]. FKBPs, including FKBP12-like proteins, exist in all domains of life. Based on the phylogenetic tree of FKBP12-like proteins we have constructed, compared to their bacterial and archaeal counterparts, plant and animal FKBP12-like proteins are highly similar within their own kingdoms in both domain architectures and sequence lengths (Figure 1).

### 3.2. OsFKBP12 Encodes a Negative Regulator of Defense Responses

Based on the presence of the conserved FKBP domain and its isolation from a defense-related subtractive cDNA library, we hypothesized that OsFKBP12 is a component of a common plant defense signaling pathway against pathogens. Therefore it is not a big leap to use transgenic *A. thaliana* as a heterologous dicot system for the rapid functional test on the effects of OsFKBP12 on plant defense responses, due to the wealth of existing knowledge in the defense signaling network in *A. thaliana* [34,35].

Phytohormones, including salicylic acid (SA) were known to play important roles in plant defense signaling [36]. *PR1* and *PR2* are two commonly used pathogenesis-related defense marker genes, primarily regulated by SA signaling and induced during the establishment of systemic acquired resistance [37,38,39,40]. The elevated expression of PR-1 protein has been demonstrated to enhance resistance towards biotrophic pathogens [38,41], and *Pst* DC3000 is a biotrophic bacterial pathogen commonly employed to test the defense response in *A. thaliana* [42,43,44]. With the ectopic over-expression of *OsFKBP12* in transgenic Arabidopsis, the induction of *PR1* and *PR2* upon pathogen inoculation was suppressed compared to the untransformed controls, which could result in increased susceptibility towards biotrophic pathogens. Meanwhile, the two *AtFKBP12-*knockout mutants had greatly enhanced expressions of *PR1* and *PR2* compared to their respective wild-type backgrounds (Figure 3C). These results were consistent with the disease phenotype as shown in Figure 3A,B, with the highest pathogen titers and most extensive lesions in the inoculation zones in the three *OsFKBP12*-transgenic lines and lowest titers and least amount of damage in the *Atfkbp12* mutants. This clearly demonstrated that *OsFKBP12* is related to plant defense responses and the overexpression of *OsFKBP12* reduced the ability of *A. thaliana* to tackle the biotrophic pathogen *Pst* DC3000.

### 3.3. OsFKBP12 Increased Salt Sensitivity in Transgenic Arabidopsis

It was previously reported that transgenic Arabidopsis ectopically expressing *PaFKBP12* from *Polytrichastrum alpinum* (sharing 73% amino acid sequence identity with OsFKBP12) exhibited enhanced salt tolerance [22]. To assess whether salt stress tolerance could be similarly enhanced by our higher-plant FKBP12 homologs, salt treatment was carried out using our *OsFKBP12*-transgenic Arabidopsis lines and the *Atfkbp12* mutants. Surprisingly, the OsFKBP12 ectopic expressors exhibited a salt-sensitive phenotype with larger extents of leaf chlorosis, lower chlorophyll contents and lowest levels of induction of the salt-inducible genes, *RD22* and *RD29a*, when compared to the wild-types (Col-0 and Col-2), while in turn, the wild types had lower salt tolerance than the two *Atfkbp12* mutants in terms of salt stress phenotypes and salt-responsive gene induction (Figure 4). This implies rice and Arabidopsis FKBP12s both act as negative regulators in abiotic stress and their function is opposite to that of their non-vascular-plant homolog. One thing to note is that it is only the second study of plant FKBP12 playing role in salt stress response and most mosses display higher tolerance to salt than ferns [45] and *P. alpinum* is one of the moss species that could survive in extreme conditions [46]. Their physiology may be quite different from that of vascular plants. In addition, there are reports showing that even if two homologous proteins share high sequence identity and have very similar protein structures, their biological activities could still be very different and even opposite to each other. This may be the result of fine-tuning of the interaction interface for ligand binding [47]. In an attempt to explain why the rice and Arabidopsis FKBP12 behave contradictory under stress condition when compared to that of PaFKBP12, we analyzed motifs of the nine closest plant FKBP12 homologues (selected from Figure 1B). With the adoption of the MEME Suite program (Version 5.2.0) (http://meme-suite.org/tools/meme) [48], four motifs were identified (Figure S4A). Amino acid residues located in the motifs that are different in the salt sensitivity conferring OsFKBP12 and AtFKBP12 when compared to the salt tolerance conferring PaFKBP12 are highlighted with red arrows (Figure S4B). As only 3 out of the 10 plant homologues are functionally characterized under salt stress, we could only speculate that some of these differences may contribute to the differential functions of different FKBP12 homologues toward salt stress. The functions of a protein rely on its activity, subcellular localization, and mobility, and these features are greatly influenced by its physio-chemical properties such as isoelectric point, phosphorylation state, and the cytosolic pH [49]. Phosphorylation sites of the three FKBP12 homologues were carried out using NetPhos 3.1 Server (http://www.cbs.dtu.dk/services/NetPhos/) [50,51]. Various phosphorylation sites were identified among the three homologues (Figure S5). In addition, theoretical isoelectric points of AtFKBP12, OsFKBP12, and PaFKBP12 are deduced as 5.74, 7.75, and 8.62, respectively, by Protparam tool (https://web.expasy.org/protparam/) [52]. The three homologues seem to possess apparently different physio-chemical properties. However, how these properties contribute to salt sensitive and resistance phenotypes remain elusive, since the manuscript is thus far the second report characterizing the functions of plant FKBP12 under salt stress.

### 3.4. OsFKBP12 Interacts with a Defense-Related GTPase, OsYchF1

Two approaches (yeast two-hybrid screening and in-vitro pull-down assay) were employed in this study to explore the protein interacting partners of OsFKBP12 in an attempt to obtain clues for the physiological role of OsFKBP12.

The identified interacting partner of OsFKBP12, OsYchF1 (BAD03576), belongs to theYchF subfamily of the Obg family of unconventional TRAFAC class of P-loop GTPases. The interaction between full-length OsYchF1 and OsFKBP12 was confirmed by in-vitro pull-down assay (Figure 5A) and in vivo BiFC experiment (Figure S3). OsYchF1 was first identified as the interacting partner of the positive defense regulator, OsGAP1, a higher plant-specific GTPase-activating protein [53]. Further studies discovered that OsYchF1 is a negative regulator of defense responses [23]. Both YchF1 and FKBP12 display high degrees of sequence identity among their respective plant homologs, suggesting that they are potentially involved in some fundamental or essential processes in plants. Since the heterologous functional test of OsFKBP12 was performed in *A. thaliana*, the interaction between OsFKBP12 and AtYchF1 was also confirmed by in-vitro pull-down assay. This provided additional evidence that the functions of FKBP12 in both rice and Arabidopsis are conserved.

Previous studies on OsYchF1 showed that OsYchF1 is involved in both biotic and abiotic stress responses as a negative regulator. In this report, OsFKBP12 is also characterized as a negative regulator in both types of stresses and identified as an interacting partner of OsYchF1. Hence, these two proteins may be involved in the same convergent pathways of biotic and abiotic stress responses in rice and Arabidopsis.

## 4. Materials and Methods

### 4.1. Plant Material and Chemicals

The rice near-isogenic lines (NILs) and their respective recurrent parents used in this study (CBB14 and its parent, SN1033, and CBB23 and its parent, JG30) were obtained previously [54]. *A. thaliana* wild type Columbia-0 (Col-0) used for transformation was a laboratory stock. Arabidopsis *AtFKBP12*-knockout mutants (CS849010 and SALK_064494) were from Arabidopsis Biological Research Centre. Enzymes and reagents for molecular and biochemical studies were obtained from Bio-Rad Laboratories (Hercules, CA, USA) and Invitrogen Corporation (Camarillo, CA, USA), unless specified otherwise. Chemicals for plant growth and tissue cultures were supplied by Sigma-Aldrich Co. (St Louis, MO, USA). Potting soil for the growth of *A. thaliana* was from Florgard Vertriebs GmbH (Gerhard-Stalling, Germany). DNA oligos were synthesized by Tech Dragon Ltd. (HKSAR, P.R.C.). Primers used are listed in Table S1.

### 4.2. Pathogen Inoculations of Rice with Xanthomonas oryza pv. oryza (Xoo) and Arabidopsis with Pseudomonas syringae pv. Tomato (Pst DC3000)

Near-isogenic rice lines harboring bacterial blight resistance genes (*Xa14* or *Xa23*) and their recurrent parents (SN1033 and JG30) were grown on regular field soil in a greenhouse (temperature 24–28 °C; RH 70–80%; under natural light). Inoculation of the *Xoo* races LN44 and P6 was performed by the clipping method when the plants were about two-month-old till the maximum tillering stage (Zhang et al., 1996). For pathogen inoculation of Arabidopsis, *Pst* DC3000 with a concentration of 10^8^ colony-forming units/mL in 10 mM MgSO_4_ supplemented with 0.02% (*v*/*v*) Silwet L-77 was inoculated into the leaf tissue via syringe infiltration when the plants were 6 weeks old [27]. After three or four days, the phenotypes of the inoculated leaves were recorded and inoculation zones were harvested as leaf discs and the titer (colony-forming units per unit area) of pathogens therein was estimated by plate count method [27].

### 4.3. Salt Treatment of Arabidopsis

For abiotic stress experiments, 10-day-old Arabidopsis seedlings were transferred onto MS agar plates (mock treatment) or MS agar plates supplemented with 150 mM NaCl (salt treatment). Chlorophyll contents were measured about 10 days after salt or mock treatment as described [55,56]. Expression levels of salt-inducible genes (*RD22* and *RD29a*) were examined by real-time RT-PCR from seedling samples harvested one day after treatment [57].

### 4.4. Cloning of OsFKBP12, and cDNA Library Construction Using Suppression Subtractive Hybridization Technique

The partial cDNA clone of *OsFKBP12* was obtained as one of the differentially expressed genes related to plant defense responses discovered previously using suppression subtractive hybridization techniques [23]. The libraries were constructed, starting with total RNA samples extracted from rice leaves of a resistant rice near-isogenic line (NIL) containing the *R* gene *Xa14* (CBB14) and its susceptible recurrent parent (SN1033) on day 4 after pathogen inoculation, using the SMART^TM^ PCR cDNA Synthesis Kit (Clontech, cat.# K1052-1) according to the manufacturer’s instructions. Subtraction libraries were then constructed in both directions (for the pair of inoculated NILs and susceptible recurrent parent) using the CLONTECH PCR-Select^TM^ cDNA Subtraction Kit (Clontech, cat.# K1804-1).

### 4.5. Phylogenetic Analyses and Sequence Alignments

Full-length sequences of OsFKBP12 and FKBP12-like proteins were retrieved from the NCBI protein database. FKBP12-like proteins were chosen from representative species in different families. Sequence alignment was performed using ClustalW algorithm. A phylogenetic tree was built with MEGA 5.05, using neighbor-joining method and 1000-bootstrap replicates.

### 4.6. Yeast Two-Hybrid, Fusion Protein Constructions and In-Vitro Pulldown Assays

Yeast two-hybrid screening experiments were carried out using the commercial kit BD Matchmaker™ Library Construction and Screening Kit (Clontech, cat.# K1615-1), according to manufacturer’s instructions. *OsFKBP12* was amplified and subcloned into the pGBKT7 vector from the full-length coding sequence (CDS) clone in the pBlueScript KSII(+) vector, with primers and PCR conditions given in Table S1. The *OsFKBP12*-containing fragment was ligated to a restriction-digested pGBKT7 vector to form in-frame fusion with the *N*-terminal c-Myc epitope tag. Other screening procedures followed as previously described [23,32].

To verify the interaction between OsFKBP12 and its interacting partner, *OsFKBP12* was amplified and subcloned into the pGEX-4T-1 vector (GE Healthcare UK Ltd., Chalfont St Giles, England) and pMAL-c2 (New England Biolabs Inc., Beverly, MA, U.S.A.) respectively, with primers and PCR conditions as stated in Table S1. The *OsFKBP12*-containing fragment was ligated to restriction-digested pGEX-4T-1 and pMAL-c2 vectors to form in-frame fusions with the *N*-terminal glutathione S-transferase (GST) and maltose-binding protein (MBP) tags respectively. Fusion proteins were expressed in *Escherichia coli* BL21/DE3 as previously described [23].

Soluble proteins were extracted from plant tissue by grinding the leaves with homogenizing buffer (20mM Tris (pH7.5), 150mM NaCl), followed by centrifugation at 16,300× *g* for 10 min, and the supernatant containing the soluble proteins were retained. For in-vitro pull-down of native rice protein, total protein was extracted from the Aichi Asahi cultivar by grinding 1 g of leaf tissue in 1 mL ice-cold immunoprecipitation buffer (50mM Tris/HCl (pH7.5), 250mM NaCl, 2 m M MgCl_2_, 0.5mM CaCl_2_, 10% (*v*/*v*) glycerol, 1.5% (*v*/*v*) Triton X-100, 1 mM PMSF, 2 μg/mL leupeptin). The mixture was centrifuged at 16,300× *g* for 10 min, and the supernatant containing the protein extract was retained. Protein quantitation was conducted with Bio-Rad Protein Assay Dye Reagent Concentrate (#5000006; Bio-Rad Laboratories, CA, USA) following the product manual. GST-tagged fusion proteins and MBP-tagged fusion proteins were extracted by MagneGST^TM^ Protein Purification System (V8600; Promega Biosciences, CA, USA) and SpinClean™ MBP Excellose^®^ Spin Kit (23020; Mbiotech, Inc., Haman, Korea), according to manual’s instructions. Protein quantitation was conducted with Bio-Rad Protein Assay Dye Reagent Concentrate as above.

All in-vitro pull-down assays were performed using MagneGST^TM^ Glutathione Particles (Promega Biosciences, V8600) following the manufacturer’s protocol. The interaction complexes were eluted by 50 mM Glutathione solution and was analyzed by western blotting and immunodetection of either the GST or MBP tag using anti-GST and anti-MBP antibodies respectively (Sigma-Aldrich, G7781 and M6295, respectively) in 12.5% gel. For the in-vitro pull-down of native OsYchF1 by GST-FKBP12, 500 μg soluble protein extracted from one-gram rice leaves were incubated with *E. coli* BL221 cell lysate containing approximately 100 μg GST-FKBP12 for 1 h with gentle shaking. The protein mixture was then loaded onto the MagneGST^TM^ Glutathione Particles for half an hour. The interaction complex was washed five times with Buffer A (20mM Tris pH7.5, 150mM NaCl). For the in-vitro pull-down of MBP-OsFKBP12 by GST-AtYchF1, *E. coli* BL221 cell lysate containing approximately 100 μg MBP-OsFKBP12 was incubated with *E. coli* BL221 cell lysate containing approximately 100 μg GST-AtYchF1 for 1 h with gentle shaking. The protein mixture was loaded onto the MagneGST^TM^ Glutathione Particles for half an hour. The interaction complex was washed 10 times with Buffer A. For the in-vitro pull-down of MBP- OsFKBP12 by GST-OsYchF1 G domain, *E. coli* BL221 cell lysate containing approximately 100 μg MBP-His-OsFKBP12 was incubated with *E. coli* BL221 cell lysate containing approximately 100 μg of GST-OsYchF1 G domain or GST-OsYchF1 TGS domain for 1 h with gentle shaking. The protein mixture was loaded to the MagneGST^TM^ Glutathione Particles for half an hour. The interaction complex was washed 10 times with Buffer A. GST tag-only and MBP tag-only were included as negative controls respectively.

### 4.7. Bimolecular Fluorescence Complementation (BiFC) Experiment

Bimolecular fluorescent complementation (BiFC) transient assay was conducted according to an established method [58]. Expression cassettes either containing C-terminal-half of cyan fluorescent protein (cCFP) or *N*-terminal-half of yellow fluorescent protein (nYFP) inserted in between cauliflower mosaic virus 35S promoter and terminator were adopted. To enhance the transformation efficiency of polyethylene glycol (PEG)-mediated DNA transfer, the cassettes were subcloned into the vector pBluescript KSII (+) for transient expression. Constructs of cCFP-OsYchF1, cCFP and nYFP were described in our previous publication [32]. Fusion construct of nYFP-OsFKBP12 was made by inserting PCR-amplified OsFKBP12 full-length cDNA in frame to the nYFP expression cassette (see Table S1 for primers and PCR conditions). Protoplast preparation and PEG-mediated transformation was adopted from a method described previously [58]. In brief, 3 day-old tobacco BY-2 culture cells were used as the starting materials. Protoplasts were obtained by digestion with enzyme solution for 3 h and transformed with 10 µg plasmid DNA of each recombinant construct carrying the expression cassette via PEG-mediated transformation. The transformed protoplasts were further cultivated in protoplast cultivating medium for 16 h. Fluorescent signals of protoplasts was then observed under a confocal microscope (Leica TCS SP8 Confocal Microscope System).

### 4.8. Online Bioinformatics Tools

MEME Suite (Version 5.2.0) (http://meme-suite.org/tools/meme) [48] and NetPhos 3.1 Server (http://www.cbs.dtu.dk/services/NetPhos/) [50,51] were applied to predict protein motifs and phosphorylation sites in plant FKBP12 homologues.

### 4.9. Statistical Analyses

Data were analyzed using the Statistical Package for Social Sciences (SPSS; version 15.0). Mean differences were analyzed by one-way analysis of variance (ANOVA), using the least-significant difference (LSD) or Games–Howell post hoc test.

## 5. Conclusions

Based on the functional characterization of OsFKBP12 and AtFKBP12 upon pathogen inoculation and salt treatment, OsFKBP12 and AtFKBP12 behaved as negative regulators in both biotic and abiotic stresses in higher plants, as demonstrated in both a monocot and a dicot model. Furthermore, we identified its interacting partner as an unconventional G-protein, YchF1, which, like FKBP12, is also an ancient protein and plays negative regulatory roles in both biotic and abiotic stresses. This is the first report showing FKBP12 being involved in both biotic and abiotic stress responses and interacting with the stress-related ancient unconventional G protein, YchF1.

## Supplementary Materials

Supplementary materials can be found at https://www.mdpi.com/1422-0067/21/22/8791/s1. Table S1. Primers for cloning and real-time PCR analyses. Table S2. Results of the yeast two-hybrid library screening of OsFKBP12. Table S3. Identity of the putative OsFKBP12-interacting protein partner. Figure S1. The expressions of the *OsFKBP12* transgene in 4 weeks-old seedlings were verified by reverse transcription followed by real-time PCR (real-time RT-PCR). Figure S2. Results of the yeast two-hybrid test using the AH109 yeast clone co-transformed with two plasmids, pGBKT7-*OsFKBP12* and pGADT7-Rec-*BAD03576* partial-fragment. Figure S3. Bimolecular fluorescence complementation (BiFC) experiment showing in vivo interaction between OsFKBP12 and OsYchF1. Figure S4 Motif analysis of plant FKBP12 homologues. Figure S5 Prediction of Phosphorylation sites in FKBP12 homologues from Arabidopsis (Ath; (A)), rice (Osa; (B)) and *P. alpinum* (Pal; (C)) with NetPhos 3.1 Server (http://www.cbs.dtu.dk/services/NetPhos/).
