# Supplementary material for: A Rice Immunophilin Homolog, OsFKBP12, Is a Negative Regulator of Both Biotic and Abiotic Stress Responses
Source: Int J Mol Sci. 2020 Nov 20;21(22):8791. doi: 10.3390/ijms21228791 (PMC7699956; doi:10.3390/ijms21228791)
Supplement: Supplementary file 1 [file ijms-21-08791-s001.pdf]

Supplementary Materials

Table S1. Primers for cloning and real-time PCR analyses.

**Primers Application**	**Sequence (5' to 3')**
For amplifying full-length *OsFKBP12* from LN44-inoculated SN1033 cDNA sample	AAGAAGAAGAAGAAGAGAGGATGG<br>GAATTACATGATGCGTTTACTGGG
For amplifying *OsFKBP12* with *Sma*I and *Sal*I restriction sites for cloning into pGBKT7 [a]	TTCCCGGGGATGGGCTTCGAGAAGACGA<br>CAGGTCGACGTTACTGGGCGCTAAGAACC
For amplifying *OsFKBP12* with *BamH*I and *Sal*I restriction sites for cloning into pGEX-4T-1 [a]	TTGGATCCCCGATGGGCTTCGAGAAGACGA<br>CAGGTCGACGTTACTGGGCGCTAAGAACC
For amplifying *OsFKBP12* with *Xho*I and *Xma*I restriction sites for cloning into KSII (+) with nYFP expression cassette [a]	TTATACTCGAGATGGGCTTCGAGAAGACGA<br>TTCCTCCCGGGCTGGGCGCTAAGAACCTC
For real-time PCR of *OsFKBP12* [b]	ACCGGCTTCGGGAAAGATAA<br>ATCACCGAACCCTGGCCTAT
For real-time PCR of *AtFKBP12* [b]	CACCGGATTCGGGAAAGATG<br>CACAGCACCTTTACCGATTTGG
For real-time PCR of *A. thaliana* polyubiquitin *UBQ10* (At4G05320) [b]	GAAGGTGCTGAGTTGATTG<br>GGACTTGACGTTGTTTGG
For realtime PCR of *A. thaliana PR1*[b]	TCAAGATAGCCCACAAGATTATC<br>CTTCTCGTTCACATAATTCCCAC
For real-time PCR of *A. thaliana PR2* [b]	ACCACCACTGATACGTCTCCTC<br>AACTTCATACTTAGACTGTCGATC
For real-time PCR of *A. thaliana RD22*[b]	GACTTTGACTCTGTTCTCGGTA<br>TTTTCCAGCTCAGCTCCT
For real-time PCR of *A. thaliana RD29a*[b]	GGGAATTCAAGGAACTTGGCAATGAA<br>GGTCTAGAGACGGATGGATCTCATTT
For real-time PCR of *OsAc1D*[b]	CTTCATAGGAATGGAAGCTGCGGGTA<br>GACCACCTTGATCTTCATGCTGCTA

[a] PCR for subcloning of *OsFKBP12* was performed as follows: 95°C for 5 min; 35 cycles of 95°C for 5 min, 55°C for 30 s, 72°C for 1 min; a final extension polymerization step at 72°C for 10 min. PCR product was resolved by gel electrophoresis on a 2% agarose gel. A single and unique band of the expected size was purified and subcloned into designated vectors via restriction digestion.

[b] Real-time PCR protocol: 95°C for 5 min; 40 cycles of 95°C for 10 s, 60°C for 30 s.

Table S2. Results of the yeast two-hybrid library screening of OsFKBP12.

	No. of colonies grown up on SD/-3	No. of colonies grown up on SD/-4	No. of positive result in colony-lift assay
1st round screening	113	92	34
2nd round screening	121	48	23

Table S3. Identity of the putative OsFKBP12-interacting protein partner.

OsFKBP12 partner clone identity	Number of hits
GTP-binding protein<br>(GenBank accession number: BAD03576, NP_001061206)	4

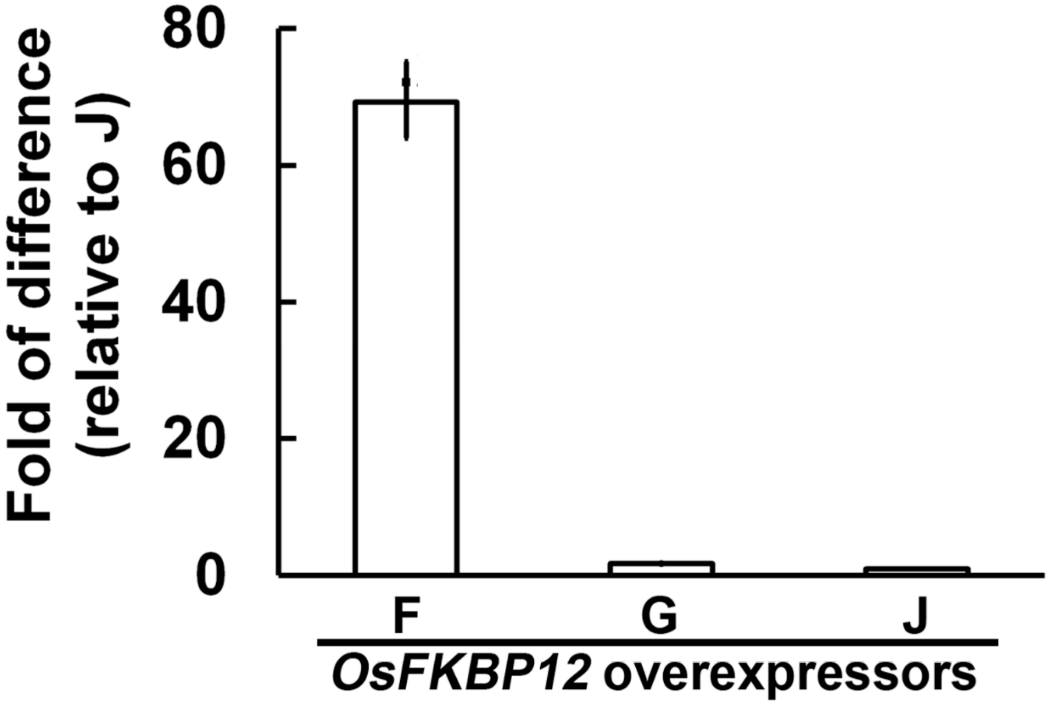

Figure S1. The expressions of the *OsFKBP12* transgene in 4 weeks-old seedlings were verified by reverse transcription followed by real-time PCR (real-time RT-PCR). The expression level of *OsFKBP12* in the transgenic line J was the lowest and was set to 1 for comparison with the transgene expression levels in the other two transgenic lines. Relative gene expression was calculated by the $2^{-\Delta\Delta CT}$ method [1]and normalized against the expression level of the polyubiquitin gene *UBQ10* [2,3].

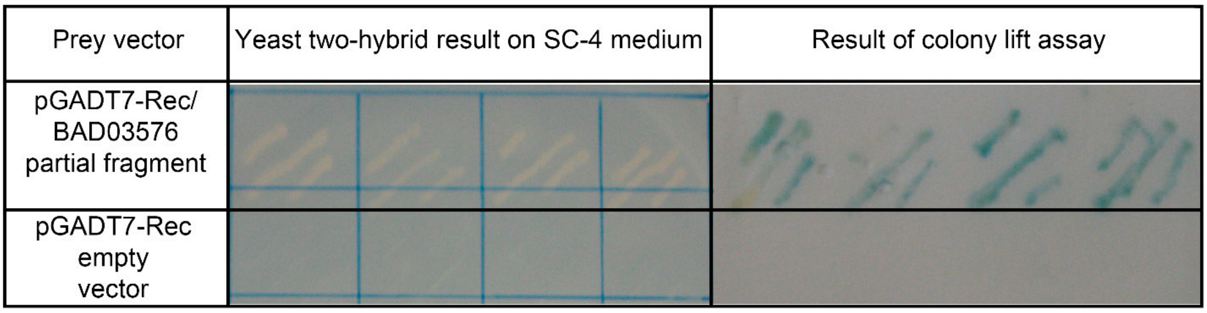

Figure S2. Results of the yeast two-hybrid test using the AH109 yeast clone co-transformed with two plasmids, pGBKT7-*OsFKBP12* and pGADT7-Rec-*BAD03576* partial-fragment, grown on SD medium lacking Trp, Leu, Ade and His (panels on the left) and colony lift assay (panels on the right). Co-transformation with pGBKT7-*OsFKBP12* and pGADT7-Rec empty vector was used as negative control. Each experiment is shown with four replicates with similar results.

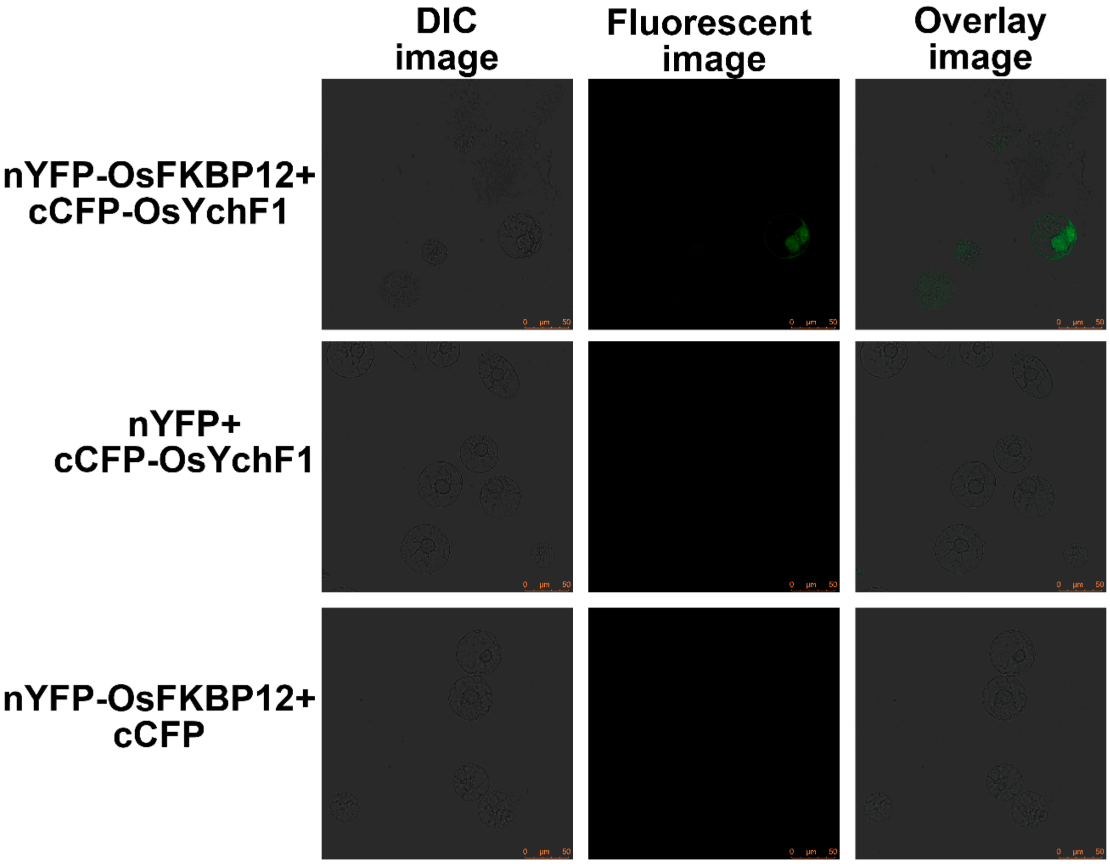

Figure S3. Bimolecular fluorescence complementation (BiFC) experiment showing *in vivo* interaction between OsFKBP12 and OsYchF1. Fluorescent signals were analyzed with confocal microscopy when fusion constructs of *nYFP-OsFKBP12* and *cCFP-OsYchF1, nYFP* and *cCFP-OsYchF1* or *nYFP-OsFKBP12* and *cCFP* were co-transfected into tobacco BY-2 protoplasts by polyethylene glycol (PEG) treatment. Differential interference contrast (DIC) images, fluorescent images and overlay images were shown. Scale bar: 50 µm. Expression cassettes were subcloned into the vector pBluescript KSII (+) for transient expression. Protoplast preparation and PEG transformation was adopted from method described in Tian *et al*. [4]. In brief, 3 days-old BY-2 culture was used as the starting materials. Protoplasts were digested with enzyme solution for 3 hours and transformed with 10 µg plasmid DNA of each construct carrying the expression cassettes via polyethylene glycol (PEG)-mediated DNA transfer, the transformed protoplast was further cultivated in protoplast cultivating medium for further 16 h. Protoplasts were then observed under confocal microscope (Leica TCS SP8 Confocal Microscope System). Constructs of *cCFP-OsYchF1*, cCFP and nYFP were described in our previous publication [5]. Fusion construct of *nYFP-OsFKBP12* was made by inserting OsFKBP12 full-length cDNA into the nYFP expression cassette in frame.

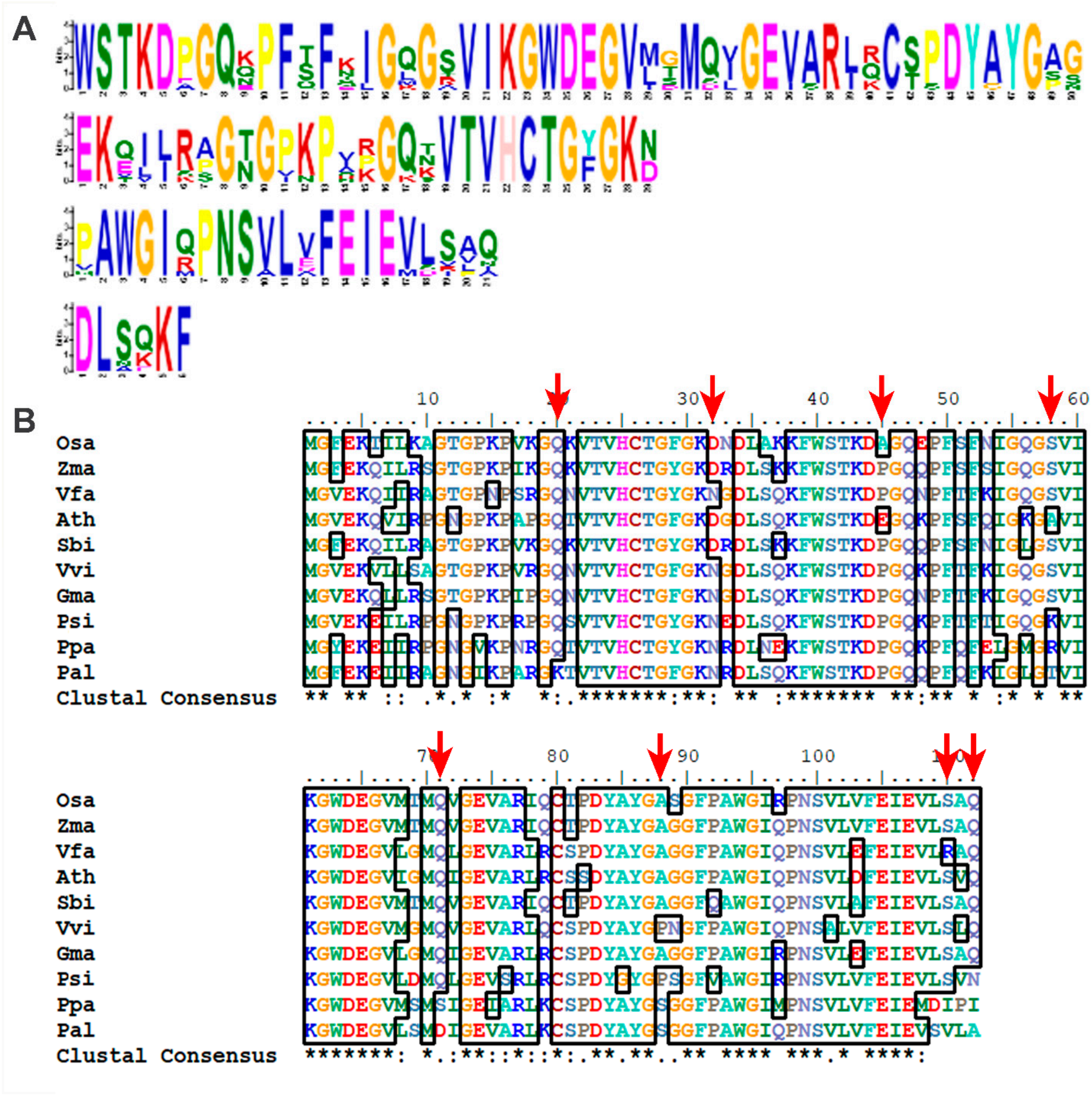

Figure S4 Motif analysis of plant FKBP12 homologues. Nine plant FKBP12 homologues closest to OsFKBP12 were selected from Figure 1B. Motif analysis was performed using the MEME Suite (Version 5.2.0) (http://meme-suite.org/tools/meme) [6]. (A) Four motifs were identified. (B) Protein sequence of the 10 plant FKBP12 homologues were aligned withClustalW [7]. "*": conserved residues; ":": conserved substitutions; and ".": semi-conserved substitutions. The three motifs identified are underlined. Osa, *O. sativa* (NP_001048188); Zma, *Z. mays* (NP_001105537); Vfa, *V. faba* (AAB57848); Ath, *A. thaliana* (NP_201240); Sbi, *Sorghum bicolor* (XP_002454586);

Vvi, *Vitis vinifera* (XP_002263647); Gma, *Glycine max* (ACU15318); Psi, *Picea sitchensis* (ABK22086); Ppa, *Physcomitrella patens* subsp. patens (XP_001756105); Pal, *Polytrichastrum alpinum* [8]. Red arrows highlighted amino acid residues located in motifs predicted in panel A and are different in the salt sensitivity conferring OsFKBP12 and AtFKBP12 homologues when compared to the salt tolerance conferring PaFKBP12.

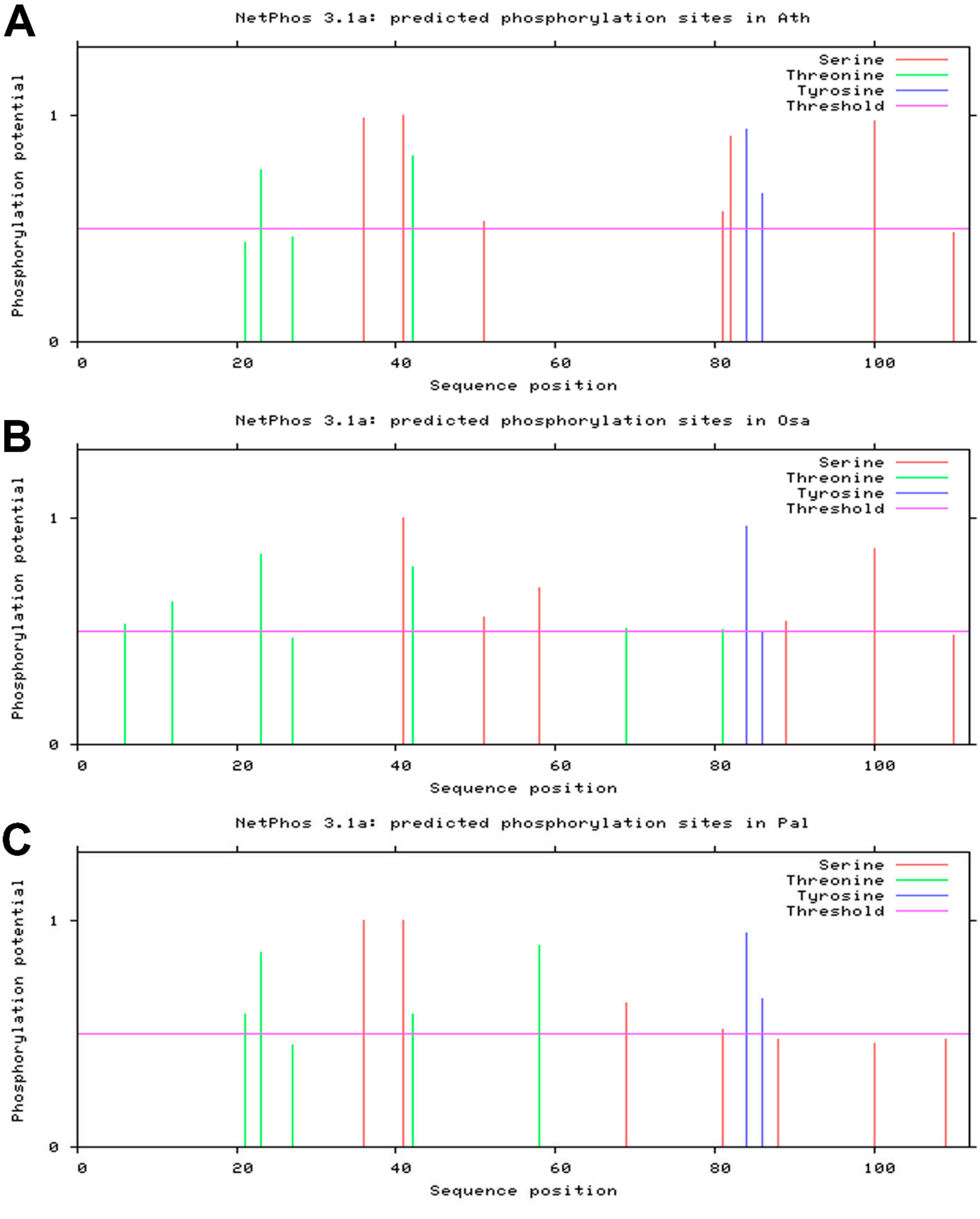

Figure S5 Prediction of Phosphorylation sites in FKBP12 homologues from Arabidopsis (Ath; (A)), rice (Osa; (B)) and *P. alpinum* (Pal; (C)) with NetPhos 3.1 Server (http://www.cbs.dtu.dk/services/NetPhos/) [9,10]. Phosphorylation potential at 0.5 was set as threshold. The higher the score, the higher confidence as a phosphorylation site.

References for Supplementary Materials

1. Livak, K.J.; Schmittgen, T.D. Analysis of relative gene expression data using real-time quantitative PCR and the 2-ΔΔCT Method. *Methods* **2001**, *25*, 402–408.
2. Czechowski, T.; Bari, R.P.; Stitt, M.; Scheible, W.R.; Udvardi, M.K. Real time RT PCR profiling of over 1400 Arabidopsis transcription factors: unprecedented sensitivity reveals novel root and shoot specific genes. *Plant J.* **2004**, *38*, 366–379.
3. Jain, M.; Nijhawan, A.; Tyagi, A.K.; Khurana, J.P. Validation of housekeeping genes as internal control for studying gene expression in rice by quantitative real-time PCR. *Biochem. Biophys. Res. Commun.* **2006**, *345*, 646–651.
4. Tian, L.; Chou, H.L.; Zhang, L.; Hwang, S.K.; Starkenburg, S.R.; Doroshenk, K.A.; Kumamaru, T.; Okita, T.W. Rna-binding protein rbp-p is required for glutelin and prolamine mrna localization in rice endosperm cells[open]. *Plant Cell* **2018**, *30*, 2529–2552, doi:10.1105/tpc.18.00321.
5. Cheung, M.Y.; Xue, Y.; Zhou, L.A.; Li, M.W.; Sun, S.S.M.; Lam, H.M. An Ancient P-Loop GTPase in Rice Is Regulated by a Higher Plant-specific Regulatory Protein. *J. Biol. Chem.* **2010**, *285*, 37359–37369.
6. Bailey, T.L.; Elkan, C. Fitting a mixture model by expectation maximization to discover motifs in biopolymers. In Proceedings of the Proceedings of the Second International Conference on Intelligent Systems for Molecular Biology; 1994; pp. 28–36.
7. Thompson, J.D.; Higgins, D.G.; Gibson, T.J. CLUSTAL W: improving the sensitivity of progressive multiple sequence alignment through sequence weighting, position-specific gap penalties and weight matrix choice. *Nucleic Acids Res.* **1994**, *22*, 4673–4680.
8. Alavilli, H.; Lee, H.; Park, M.; Yun, D.J.; Lee, B. ha Enhanced multiple stress tolerance in Arabidopsis by overexpression of the polar moss peptidyl prolyl isomerase FKBP12 gene. *Plant Cell Rep.* **2018**, *37*, 453–465, doi:10.1007/s00299-017-2242-9.
9. Blom, N.; Sicheritz-Pontén, T.; Gupta, R.; Gammeltoft, S.; Brunak, S. Prediction of post-translational glycosylation and phosphorylation of proteins from the amino acid sequence. *Proteomics* **2004**, *4*, 1633–1649, doi:10.1002/pmic.200300771.
10. Blom, N.; Gammeltoft, S.; Brunak, S. Sequence and structure-based prediction of eukaryotic protein phosphorylation sites. *J. Mol. Biol.* **1999**, *294*, 1351–1362, doi:10.1006/jmbi.1999.3310.
